# *cis*-Dichlorido(1,3-dimesitylimidazolidin-2-yl­idene)(2-formyl­benzyl­idene-κ^2^ *C*,*O*)ruthenium diethyl ether solvate

**DOI:** 10.1107/S1600536810000826

**Published:** 2010-01-13

**Authors:** Christian Slugovc, Bernhard Perner, Franz Stelzer, Kurt Mereiter

**Affiliations:** a Institute for Chemistry and Technology of Materials (ICTM), Graz University of Technology, Stremayrgasse 16, A-8010 Graz, Austria; b Institute of Chemical Technologies and Analytics, Vienna University of Technology, Getreidemarkt 9/164SC, A-1060 Vienna, Austria

## Abstract

The title compound, [RuCl_2_(C_8_H_6_O)(C_21_H_26_N_2_)]·C_4_H_10_O, contains a catalytically active ruthenium carbene complex of the ‘second-generation Grubbs/Hoveyda’ type with Ru in a square-pyramidal coordination, the apex of which is formed by the benzyl­idene carbene atom with Ru=C 1.827 (2) Å. The complex shows the uncommon *cis*, rather than the usual *trans*, arrangement of the two chloride ligands, with Ru—Cl bond lengths of 2.3548 (6) and 2.3600 (6) Å, and a Cl—Ru—Cl angle of 89.76 (2)°. This *cis* configuration is desirable for certain applications of ring-opening metathesis polymerization (ROMP) of strained cyclic olefins. The crystalline solid is a diethyl ether solvate, which is built up from a porous framework of Ru complexes held together by π–π stacking and C—H⋯Cl and C—H⋯O inter­actions. The disordered diethyl ether solvent mol­ecules are contained in two independent infinite channels, which extend parallel to the *c* axis at *x*,*y* = 0,0 and *x*,*y* = 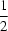,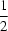 and have solvent-accessible void volumes of 695 and 464 Å^3^ per unit cell.

## Related literature

For the synthesis and application of the title compound in ring-opening metathesis polymerization (ROMP), see: Slugovc *et al.* (2004 ▶); Burtscher *et al.* (2006 ▶). For thermally switchable initiators for olefin metathesis polymerization, see: Gstrein *et al.* (2007 ▶); Szadkowska & Grela (2008 ▶). For a recent authoritative review on ruthenium-based heterocyclic carbene-coordinated olefin metathesis catalysts, see: Vougioukalakis & Grubbs (2010 ▶).
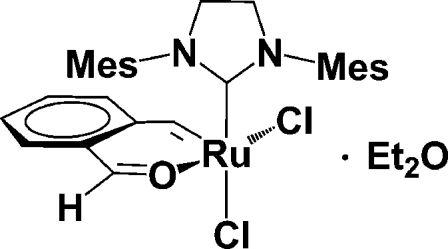


## Experimental

### 

#### Crystal data

[RuCl_2_(C_8_H_6_O)(C_21_H_26_N_2_)]·C_4_H_10_O
*M* *_r_* = 670.66
Tetragonal, 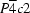

*a* = 19.8603 (4) Å
*c* = 15.6582 (7) Å
*V* = 6176.1 (3) Å^3^
*Z* = 8
Mo *K*α radiation
μ = 0.71 mm^−1^
*T* = 100 K
0.43 × 0.25 × 0.22 mm

#### Data collection

Bruker SMART APEX CCD diffractometer
Absorption correction: multi-scan (*SADABS*; Bruker, 2003 ▶) *T*_min_ = 0.78, *T*_max_ = 0.86
90504 measured reflections
8992 independent reflections
7306 reflections with *I* > 2σ(*I*)
*R*_int_ = 0.058

#### Refinement

*R*[*F* ^2^ > 2σ(*F* ^2^)] = 0.029
*wR*(*F* ^2^) = 0.067
*S* = 1.01
8992 reflections
322 parameters
H-atom parameters constrained
Δρ_max_ = 0.42 e Å^−3^
Δρ_min_ = −0.31 e Å^−3^
Absolute structure: Flack (1983 ▶), 4175 Friedel pairs
Flack parameter: −0.02 (2)

### 

Data collection: *SMART* (Bruker, 2003 ▶); cell refinement: *SAINT* (Bruker, 2003 ▶); data reduction: *SAINT*, *SADABS* and *XPREP* (Bruker, 2003 ▶); program(s) used to solve structure: *SHELXS97* (Sheldrick, 2008 ▶); program(s) used to refine structure: *SHELXL97* (Sheldrick, 2008 ▶) and *PLATON* (Spek, 2009 ▶); molecular graphics: *SHELXTL* (Sheldrick, 2008 ▶) and *Mercury* (Macrae *et al.*, 2006 ▶); software used to prepare material for publication: *SHELXL97*, *enCIFer* (Allen *et al.*, 2004 ▶), *PLATON* and *publCIF* (Westrip, 2010 ▶).

## Supplementary Material

Crystal structure: contains datablocks global, I. DOI: 10.1107/S1600536810000826/bt5164sup1.cif

Structure factors: contains datablocks I. DOI: 10.1107/S1600536810000826/bt5164Isup2.hkl

Additional supplementary materials:  crystallographic information; 3D view; checkCIF report

## Figures and Tables

**Table 1:** Hydrogen-bond geometry (Å, °)

*D*—H⋯*A*	*D*—H	H⋯*A*	*D*⋯*A*	*D*—H⋯*A*
C29—H29*C*⋯Cl1	0.98	2.67	3.634 (3)	166
C39—H39*C*⋯Cl1	0.98	2.76	3.371 (3)	121
C37—H37*A*⋯O49	0.98	2.39	3.268 (3)	150
C13—H13*B*⋯Cl1^i^	0.99	2.94	3.395 (2)	109
C14—H14*A*⋯Cl1^i^	0.99	2.90	3.324 (3)	107
C27—H27*A*⋯Cl2^i^	0.98	2.85	3.724 (3)	149
C37—H37*C*⋯Cl2^i^	0.98	2.88	3.736 (3)	146
C25—H25⋯Cl1^ii^	0.95	2.98	3.831 (3)	150
C29—H29*A*⋯Cl1^ii^	0.98	2.71	3.673 (3)	169
C46—H46⋯Cl2^iii^	0.95	3.04	3.553 (2)	115
C48—H48⋯O49^iii^	0.95	2.50	3.014 (3)	114

Symmetry codes: (i) 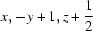; (ii) 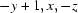; (iii) 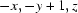.

**Fig. 1.**
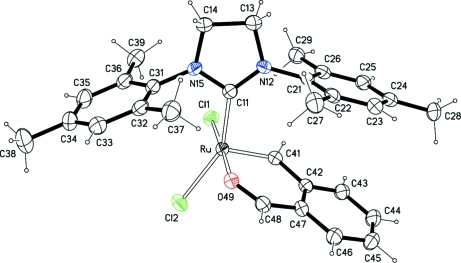
The structure of (I) with displacement ellipsoids for the non-hydrogen atoms drawn at the 50% probability level.

**Fig. 2.**
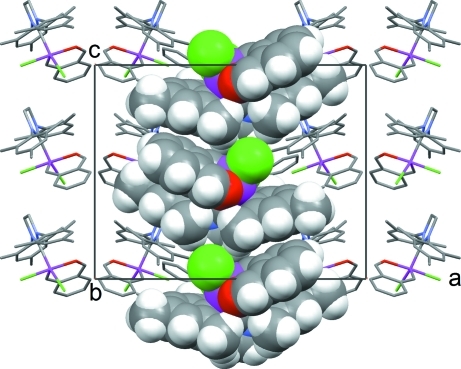
Packing diagram of (I) viewed along the b-axis. The Ru complexes in the center are shown in space-filling representation in order to emphasize their column-like stacking along the c-axis and part of their π-π stacking interactions.

**Fig. 3.**
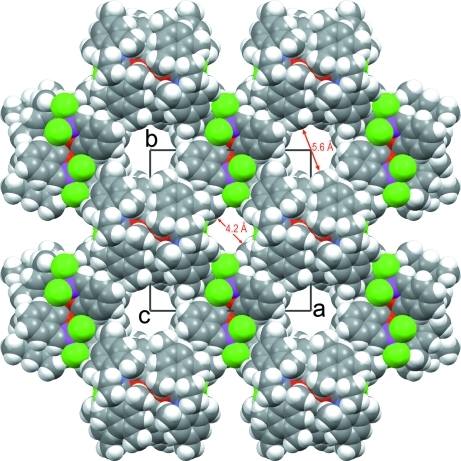
Packing diagram of (I) viewed down the c-axis showing the two different kinds of channels which are occupied by disordered diethyl ether molecules.

## supplementary crystallographic information

### Comment

The ruthenium complex RuCl_2_(C_8_H_6_O)(C_21_H_26_N_2_), which is the main constituent of the title compound, (I), was prepared by a carbene exchange reaction of (H_2_IMes)(pyridine)_2_(Cl)_2_RuCHPh (1eq.; H_2_IMes = 1,3-bismesityl-4,5-dihydroimidazol-2-ylidene) with 2-vinylbenzaldehyde (2 eq.) in CH_2_Cl_2_ at room temperature (Slugovc *et al.*, 2004). In sharp contrast to most of the ruthenium carbene complexes bearing two halides and neutral donor co-ligands (phosphines or N-heterocyclic carbenes), which exhibit a *trans* stereochemistry of the two halide ligands, the ruthenium complex of the title compound bears them in a *cis*-disposition of a square pyramidal coordination about Ru, the apex of which is formed by the benzylidene carbon C41 with a characteristically short Ru—C bond of 1.827 (2) Å whereas the bond to the N-heterocyclic carbene carbon C11 is longer by 0.077 Å (Fig. 1 and Table 1). It has been shown, that *cis*-isomer is thermodynamically favoured over its *trans*-dichlorido counterpart (Slugovc *et al.*, 2004). Ruthenium carbene complexes bearing a *cis*-dichlorido arrangement are particularly interesting, because they exhibit distinctly lower initiation rates in ring opening metathesis polymerization (ROMP) of strained cyclic olefins when compared to their *trans*-dichlorido counterparts (Gstrein *et al.*, 2007). This feature is used to design latent ROMP initiators and catalysts for *e.g.* ring closing metathesis at elevated temperatures (Szadkowska & Grela, 2008; Burtscher *et al.*, 2006; Vougioukalakis & Grubbs, 2010).

A view of the Ru complex in the title compound is presented in Fig. 1. Bond lengths and angles about Ru (Table 1) are in good agreement with the bis-dichloromethane solvate of the same complex, RuCl_2_(C_8_H_6_O)(C_21_H_26_N_2_).2CH_2_Cl_2_, which crystallizes in a moclinic lattice, space group *P*2_1_/c, *a* = 12.1933 (6), *b* = 15.4520 (7), *c* = 19.3799 (9) Å, *β* = 108.181 (1)°, *V* = 3469.1 (3) Å^3^, *Z* = 4 (Slugovc *et al.*, 2004). Both complexes, in (I) and in the dichloromethane solvate, show similar conformations and are stabilized by significant intramolecular π-π stacking interactions between the 2-formylbenzylidene and the adjacent mesityl moiety with the shortest intramolecular π-π contacts of C41···C21 = 3.00 Å, C42···C22 = 3.40 Å, and C43···C24 = 3.45 Å in (I) and 2.99, 3.42, and 3.43 Å in the dichloromethane solvate. Moreover, both complexes show intramolecular C—H···O,Cl interactions, *e.g.* in (I) between C37 and Cl1 and and C29 and Cl1 (Fig. 1 and Table 2). In contrast to the dichloromethane solvate, where the Ru complexes do not show any intermolecular π-π-stacking but are held together mainly by C—H···π and C—H···Cl intercations, intermolecular π-π-stacking is an important factor in the crystal structure of (I). Fig. 2 demonstrates that the structure of (I) contains columnar stacks of molecules extending along the *c*-axis and showing intermolecular π-π-stacking between the formylbenzylidene and one of the two mesityl groups [corresponding π-π-contacts are C44···C33(*x*,1 - *y*,-1/2 + *z*) = 3.59 Å and C43···C32(*x*,1 - *y*,-1/2 + *z*) = 3.81 Å]. Further π-π-stacking interactions arise from the mutual indentation of these stacks [corresponding π-π-contacts are C22···C24(*y*,*x*,1/2 - *z*) = 3.82 Å, C23···C23(*y*,*x*,1/2 - *z*) = 3.64 Å and C24···C22(*y*,*x*,1/2 - *z*) = 3.82 Å]. Finally, the Ru-complexes are also held together by a larger number of weak intermolecular C—H···Cl,*O* interactions (Table 2). The result of all these interactions between the Ru complexes in (I) is a framework-like structure of tetragonal symmetry containing continuous channels which extend along the *c*-axis and contain the diethyl ether solvent molecules. As shown in Fig. 3, there are two different kinds of continuous channels in the this framework, both coinciding with the two crystallographically different sets of 4 axes of the lattice. The larger channel in this framework is centered at *x,y* = 0,0 and has a minimal net-diameter in the (001)-projection of 5.6 Å and a solvent-accessible volume per unit cell of 695 Å^3^ (program *PLATON*; Spek, 2009). The smaller channel is centered at *x,y* = 1/2,1/2, has in the projection a minimal net-diameter of 4.2 Å and a solvent-accessible volume per unit cell of 464 Å^3^. As described in the experimental section, the diethyl ether solvent molecules inside these channels are disordered with about 5 molecules per unit cell in the large and about 3 molecules per unit cell in the small channel.

### Experimental

The title compound was synthesized as described by Slugovc *et al.* (2004). It was then dissolved in a small amount of CHCl_3_ and crystallized at room temperature by the vapour diffusion method using diethyl ether as the anti-solvent. Small green prismatic crystals were obtained, which remained stable at room temperature under oil for at least one hour. They were accompanied by some larger green crystals of different morphology, which after removal from the mother liquor crumbled by solvent loss within minutes and were probably a CHCl_3_ containing solvate.

### Refinement

All H atoms were placed in calculated positions and thereafter treated as riding. A torsional parameter was refined for each methyl group. *U*_iso_(H) = 1.2*U*_eq_(C_non-methyl_) and *U*_iso_(H) = 1.5*U*_eq_(C_methyl_) were used. The diethyl ether solvent molecules, which reside in two different infinite channels extending about the 4 axes parallel to the *c*-axis were disordered. The presence of CHCl_3_ was ruled out because solvent Fourier peaks did not exceed 2.2 e Å^-3^ in height. The solvent was initially approximated by 10 partly occupied carbon positions, which indicated the presence of about 4.7 diethyl ether molecules per unit cell in the larger and about 3.2 molecules per unit cell in the smaller channel. The solvent accessible void volumes of the two channels were 695 and 464 Å^3^ per unit cell (program *PLATON*; Spek, 2009). In the final refinement the solvent peaks were omitted and the contribution of the solvent to the structure factors was removed with procedure *SQUEEZE* of program *PLATON* (version-250809; Spek, 2009). Chemical formula and quantities derived thereof are given in the crystal data for an idealized solvent content of 1 molecule of diethyl ether per formula unit.

### Crystal data

**Table:**

[RuCl_2_(C_8_H_6_O)(C_21_H_26_N_2_)]·C_4_H_10_O	*D*_x_ = 1.443 Mg m^−^^3^
*M**_r_* = 670.66	Mo *K*α radiation, λ = 0.71073 Å
Tetragonal, *P*4*c*2	Cell parameters from 8875 reflections
*a* = 19.8603 (4) Å	θ = 2.3–29.6°
*c* = 15.6582 (7) Å	µ = 0.71 mm^−^^1^
*V* = 6176.1 (3) Å^3^	*T* = 100 K
*Z* = 8	Prism, green
*F*(000) = 2784	0.43 × 0.25 × 0.22 mm

### Data collection

**Table:**

Bruker SMART APEX CCD diffractometer	8992 independent reflections
Radiation source: normal-focus sealed tube	7306 reflections with *I* > 2σ(*I*)
graphite	*R*_int_ = 0.058
φ and ω scans	θ_max_ = 30.0°, θ_min_ = 2.6°
Absorption correction: multi-scan (*SADABS*; Bruker, 2003)	*h* = −27→27
*T*_min_ = 0.78, *T*_max_ = 0.86	*k* = −27→27
90504 measured reflections	*l* = −21→21

### Refinement

**Table:**

Refinement on *F*^2^	Secondary atom site location: difference Fourier map
Least-squares matrix: full	Hydrogen site location: inferred from neighbouring sites
*R*[*F*^2^ > 2σ(*F*^2^)] = 0.029	H-atom parameters constrained
*wR*(*F*^2^) = 0.067	*w* = 1/[σ^2^(*F*_o_^2^) + (0.0309*P*)^2^ + 1.9302*P*] where *P* = (*F*_o_^2^ + 2*F*_c_^2^)/3
*S* = 1.01	(Δ/σ)_max_ = 0.001
8992 reflections	Δρ_max_ = 0.42 e Å^−^^3^
322 parameters	Δρ_min_ = −0.31 e Å^−^^3^
0 restraints	Absolute structure: Flack (1983), 4175 Friedel pairs
Primary atom site location: structure-invariant direct methods	Flack parameter: −0.02 (2)

### Special details

**Table:**

Geometry. All e.s.d.'s (except the e.s.d. in the dihedral angle between two l.s. planes) are estimated using the full covariance matrix. The cell e.s.d.'s are taken into account individually in the estimation of e.s.d.'s in distances, angles and torsion angles; correlations between e.s.d.'s in cell parameters are only used when they are defined by crystal symmetry. An approximate (isotropic) treatment of cell e.s.d.'s is used for estimating e.s.d.'s involving l.s. planes.
Refinement. Refinement of *F*^2^ against ALL reflections. The weighted *R*-factor *wR* and goodness of fit *S* are based on *F*^2^, conventional *R*-factors *R* are based on *F*, with *F* set to zero for negative *F*^2^. The threshold expression of *F*^2^ > σ(*F*^2^) is used only for calculating *R*-factors(gt) *etc*. and is not relevant to the choice of reflections for refinement. *R*-factors based on *F*^2^ are statistically about twice as large as those based on *F*, and *R*- factors based on ALL data will be even larger.

### Fractional atomic coordinates and isotropic or equivalent isotropic displacement parameters (Å^2^)

**Table:**

	*x*	*y*	*z*	*U*_iso_*/*U*_eq_	
Ru	0.163541 (9)	0.497964 (9)	0.046582 (10)	0.02129 (4)	
Cl1	0.27041 (3)	0.53239 (3)	−0.00175 (4)	0.02852 (11)	
Cl2	0.11254 (3)	0.57173 (3)	−0.05230 (4)	0.03066 (12)	
C11	0.20523 (12)	0.48129 (11)	0.16118 (14)	0.0236 (5)	
N12	0.23047 (10)	0.42655 (10)	0.19933 (12)	0.0262 (4)	
C13	0.25911 (14)	0.44070 (13)	0.28464 (16)	0.0346 (6)	
H13A	0.3083	0.4328	0.2854	0.042*	
H13B	0.2376	0.4125	0.3291	0.042*	
C14	0.24273 (15)	0.51526 (12)	0.29779 (18)	0.0345 (6)	
H14A	0.2094	0.5216	0.3442	0.041*	
H14B	0.2838	0.5415	0.3111	0.041*	
N15	0.21431 (10)	0.53461 (9)	0.21391 (12)	0.0262 (4)	
C21	0.23113 (12)	0.35894 (11)	0.16561 (14)	0.0253 (5)	
C22	0.17748 (12)	0.31643 (12)	0.18503 (14)	0.0269 (5)	
C23	0.17985 (12)	0.25113 (12)	0.15293 (15)	0.0288 (5)	
H23	0.1435	0.2214	0.1646	0.035*	
C24	0.23375 (13)	0.22794 (12)	0.10419 (16)	0.0302 (5)	
C25	0.28701 (12)	0.27169 (12)	0.08777 (16)	0.0296 (5)	
H25	0.3243	0.2562	0.0553	0.035*	
C26	0.28676 (12)	0.33789 (12)	0.11807 (15)	0.0285 (5)	
C27	0.11903 (14)	0.33958 (13)	0.23832 (17)	0.0348 (6)	
H27A	0.1354	0.3547	0.2941	0.052*	
H27B	0.0874	0.3022	0.2462	0.052*	
H27C	0.0962	0.3769	0.2094	0.052*	
C28	0.23392 (14)	0.15699 (12)	0.06930 (18)	0.0359 (6)	
H28A	0.2786	0.1464	0.0464	0.054*	
H28B	0.2004	0.1532	0.0237	0.054*	
H28C	0.2229	0.1253	0.1152	0.054*	
C29	0.34414 (13)	0.38489 (13)	0.09885 (18)	0.0350 (6)	
H29A	0.3799	0.3602	0.0691	0.052*	
H29B	0.3619	0.4033	0.1524	0.052*	
H29C	0.3281	0.4218	0.0626	0.052*	
C31	0.18137 (13)	0.59830 (11)	0.20283 (15)	0.0275 (5)	
C32	0.11424 (13)	0.60492 (12)	0.22833 (15)	0.0303 (5)	
C33	0.08306 (13)	0.66723 (13)	0.21854 (17)	0.0339 (6)	
H33	0.0371	0.6719	0.2344	0.041*	
C34	0.11702 (15)	0.72254 (13)	0.18645 (17)	0.0355 (6)	
C35	0.18389 (14)	0.71537 (13)	0.16534 (17)	0.0361 (6)	
H35	0.2076	0.7535	0.1447	0.043*	
C36	0.21842 (14)	0.65417 (13)	0.17305 (16)	0.0317 (5)	
C37	0.07556 (14)	0.54747 (13)	0.26922 (17)	0.0359 (6)	
H37A	0.0719	0.5101	0.2286	0.054*	
H37B	0.0304	0.5630	0.2849	0.054*	
H37C	0.0994	0.5322	0.3205	0.054*	
C38	0.08123 (17)	0.78913 (15)	0.1766 (2)	0.0498 (8)	
H38A	0.1139	0.8259	0.1818	0.075*	
H38B	0.0470	0.7936	0.2213	0.075*	
H38C	0.0596	0.7911	0.1204	0.075*	
C39	0.29151 (14)	0.64977 (14)	0.15142 (18)	0.0384 (6)	
H39A	0.3106	0.6089	0.1767	0.058*	
H39B	0.3150	0.6893	0.1741	0.058*	
H39C	0.2969	0.6482	0.0892	0.058*	
C41	0.17781 (11)	0.41460 (11)	0.00063 (15)	0.0247 (4)	
H41	0.2229	0.4046	−0.0152	0.030*	
C42	0.12846 (11)	0.36162 (11)	−0.01471 (14)	0.0237 (4)	
C43	0.14811 (12)	0.30477 (12)	−0.06108 (16)	0.0291 (5)	
H43	0.1929	0.3017	−0.0820	0.035*	
C44	0.10326 (13)	0.25294 (13)	−0.07703 (17)	0.0349 (6)	
H44	0.1178	0.2150	−0.1091	0.042*	
C45	0.03799 (13)	0.25513 (12)	−0.0475 (2)	0.0382 (6)	
H45	0.0079	0.2189	−0.0584	0.046*	
C46	0.01669 (12)	0.31127 (12)	−0.00132 (19)	0.0329 (5)	
H46	−0.0283	0.3135	0.0191	0.039*	
C47	0.06097 (12)	0.36413 (11)	0.01500 (15)	0.0254 (5)	
C48	0.03578 (12)	0.42013 (11)	0.06446 (14)	0.0256 (5)	
H48	−0.0097	0.4179	0.0830	0.031*	
O49	0.06864 (8)	0.47089 (8)	0.08469 (10)	0.0247 (3)	

### Atomic displacement parameters (Å^2^)

**Table:**

	*U*^11^	*U*^22^	*U*^33^	*U*^12^	*U*^13^	*U*^23^
Ru	0.02463 (9)	0.01835 (8)	0.02089 (6)	0.00016 (7)	−0.00329 (7)	0.00037 (7)
Cl1	0.0282 (3)	0.0274 (3)	0.0299 (3)	−0.0055 (2)	−0.0025 (2)	0.0014 (2)
Cl2	0.0356 (3)	0.0285 (3)	0.0279 (2)	0.0077 (2)	−0.0004 (2)	0.0049 (2)
C11	0.0246 (11)	0.0228 (10)	0.0234 (11)	−0.0010 (8)	−0.0020 (8)	0.0002 (8)
N12	0.0320 (10)	0.0237 (10)	0.0230 (8)	0.0054 (8)	−0.0083 (8)	−0.0013 (8)
C13	0.0451 (15)	0.0300 (13)	0.0288 (13)	0.0075 (11)	−0.0136 (11)	−0.0045 (10)
C14	0.0486 (16)	0.0283 (14)	0.0265 (11)	0.0037 (10)	−0.0150 (12)	−0.0027 (11)
N15	0.0354 (11)	0.0215 (10)	0.0218 (9)	−0.0018 (8)	−0.0085 (8)	−0.0018 (7)
C21	0.0306 (12)	0.0206 (11)	0.0248 (11)	0.0079 (9)	−0.0075 (9)	−0.0015 (9)
C22	0.0309 (12)	0.0255 (11)	0.0243 (11)	0.0063 (10)	−0.0056 (9)	0.0027 (9)
C23	0.0302 (12)	0.0252 (12)	0.0309 (12)	0.0012 (10)	−0.0049 (10)	0.0026 (9)
C24	0.0339 (13)	0.0264 (12)	0.0301 (12)	0.0075 (10)	−0.0114 (10)	−0.0025 (10)
C25	0.0271 (12)	0.0280 (12)	0.0336 (13)	0.0081 (10)	−0.0057 (10)	−0.0046 (10)
C26	0.0256 (12)	0.0297 (12)	0.0304 (12)	0.0064 (9)	−0.0070 (9)	0.0008 (10)
C27	0.0401 (15)	0.0304 (14)	0.0341 (13)	0.0037 (11)	0.0033 (11)	0.0010 (10)
C28	0.0401 (15)	0.0213 (12)	0.0463 (15)	0.0069 (10)	−0.0076 (12)	−0.0069 (10)
C29	0.0273 (13)	0.0310 (13)	0.0466 (15)	0.0039 (10)	−0.0056 (11)	−0.0010 (12)
C31	0.0368 (13)	0.0207 (10)	0.0251 (10)	−0.0017 (9)	−0.0081 (10)	−0.0005 (9)
C32	0.0367 (14)	0.0260 (12)	0.0282 (12)	−0.0048 (10)	−0.0084 (10)	−0.0026 (9)
C33	0.0305 (13)	0.0312 (13)	0.0400 (15)	0.0000 (10)	−0.0047 (11)	−0.0042 (11)
C34	0.0450 (16)	0.0234 (12)	0.0381 (14)	0.0018 (11)	−0.0060 (12)	−0.0022 (10)
C35	0.0461 (16)	0.0215 (12)	0.0406 (14)	−0.0065 (11)	−0.0035 (12)	0.0008 (10)
C36	0.0393 (14)	0.0267 (12)	0.0290 (12)	−0.0050 (11)	−0.0072 (11)	−0.0034 (10)
C37	0.0401 (15)	0.0320 (14)	0.0356 (13)	−0.0036 (12)	0.0013 (11)	−0.0002 (11)
C38	0.0548 (19)	0.0303 (15)	0.064 (2)	0.0097 (13)	0.0022 (16)	0.0049 (14)
C39	0.0403 (15)	0.0334 (14)	0.0416 (14)	−0.0077 (11)	−0.0017 (12)	−0.0058 (11)
C41	0.0244 (10)	0.0235 (10)	0.0261 (11)	0.0015 (8)	−0.0037 (9)	0.0015 (9)
C42	0.0253 (11)	0.0220 (10)	0.0238 (10)	−0.0001 (9)	−0.0036 (8)	0.0035 (8)
C43	0.0281 (11)	0.0271 (12)	0.0320 (12)	0.0035 (9)	−0.0020 (10)	−0.0044 (10)
C44	0.0342 (14)	0.0253 (12)	0.0453 (14)	0.0042 (10)	−0.0057 (11)	−0.0082 (10)
C45	0.0324 (13)	0.0256 (12)	0.0566 (16)	−0.0024 (10)	−0.0102 (14)	−0.0079 (12)
C46	0.0271 (12)	0.0255 (12)	0.0461 (13)	−0.0006 (9)	−0.0040 (11)	−0.0006 (11)
C47	0.0272 (11)	0.0201 (10)	0.0289 (11)	0.0025 (8)	−0.0027 (9)	0.0024 (9)
C48	0.0240 (11)	0.0246 (11)	0.0282 (12)	0.0025 (9)	0.0000 (9)	0.0016 (9)
O49	0.0252 (8)	0.0219 (8)	0.0271 (8)	0.0016 (6)	−0.0018 (6)	0.0005 (6)

### Geometric parameters (Å, °)

**Table:**

Ru—C41	1.827 (2)	C31—C32	1.398 (4)
Ru—C11	2.004 (2)	C31—C36	1.411 (3)
Ru—O49	2.0487 (16)	C32—C33	1.392 (3)
Ru—Cl1	2.3548 (6)	C32—C37	1.517 (4)
Ru—Cl2	2.3600 (6)	C33—C34	1.383 (4)
C11—N12	1.338 (3)	C33—H33	0.9500
C11—N15	1.355 (3)	C34—C35	1.376 (4)
N12—C21	1.443 (3)	C34—C38	1.509 (4)
N12—C13	1.479 (3)	C35—C36	1.401 (4)
C13—C14	1.530 (3)	C35—H35	0.9500
C13—H13A	0.9900	C36—C39	1.493 (4)
C13—H13B	0.9900	C37—H37A	0.9800
C14—N15	1.480 (3)	C37—H37B	0.9800
C14—H14A	0.9900	C37—H37C	0.9800
C14—H14B	0.9900	C38—H38A	0.9800
N15—C31	1.435 (3)	C38—H38B	0.9800
C21—C22	1.393 (3)	C38—H38C	0.9800
C21—C26	1.396 (3)	C39—H39A	0.9800
C22—C23	1.392 (3)	C39—H39B	0.9800
C22—C27	1.502 (3)	C39—H39C	0.9800
C23—C24	1.393 (3)	C41—C42	1.458 (3)
C23—H23	0.9500	C41—H41	0.9500
C24—C25	1.393 (4)	C42—C43	1.398 (3)
C24—C28	1.511 (3)	C42—C47	1.420 (3)
C25—C26	1.398 (3)	C43—C44	1.384 (3)
C25—H25	0.9500	C43—H43	0.9500
C26—C29	1.504 (4)	C44—C45	1.377 (4)
C27—H27A	0.9800	C44—H44	0.9500
C27—H27B	0.9800	C45—C46	1.395 (4)
C27—H27C	0.9800	C45—H45	0.9500
C28—H28A	0.9800	C46—C47	1.393 (3)
C28—H28B	0.9800	C46—H46	0.9500
C28—H28C	0.9800	C47—C48	1.445 (3)
C29—H29A	0.9800	C48—O49	1.242 (3)
C29—H29B	0.9800	C48—H48	0.9500
C29—H29C	0.9800		
			
C41—Ru—C11	97.98 (9)	C26—C29—H29C	109.5
C41—Ru—O49	91.12 (8)	H29A—C29—H29C	109.5
C11—Ru—O49	94.35 (8)	H29B—C29—H29C	109.5
C41—Ru—Cl1	89.81 (7)	C32—C31—C36	121.2 (2)
C11—Ru—Cl1	87.90 (7)	C32—C31—N15	118.9 (2)
O49—Ru—Cl1	177.42 (5)	C36—C31—N15	119.7 (2)
C41—Ru—Cl2	111.76 (7)	C33—C32—C31	118.4 (2)
C11—Ru—Cl2	150.16 (7)	C33—C32—C37	119.3 (2)
O49—Ru—Cl2	87.66 (5)	C31—C32—C37	122.2 (2)
Cl1—Ru—Cl2	89.76 (2)	C34—C33—C32	121.9 (2)
N12—C11—N15	108.25 (19)	C34—C33—H33	119.0
N12—C11—Ru	133.55 (16)	C32—C33—H33	119.0
N15—C11—Ru	118.14 (16)	C35—C34—C33	118.4 (2)
C11—N12—C21	126.60 (18)	C35—C34—C38	121.4 (3)
C11—N12—C13	113.14 (19)	C33—C34—C38	120.2 (3)
C21—N12—C13	120.26 (18)	C34—C35—C36	122.8 (2)
N12—C13—C14	102.93 (18)	C34—C35—H35	118.6
N12—C13—H13A	111.2	C36—C35—H35	118.6
C14—C13—H13A	111.2	C35—C36—C31	117.1 (2)
N12—C13—H13B	111.2	C35—C36—C39	120.5 (2)
C14—C13—H13B	111.2	C31—C36—C39	122.4 (2)
H13A—C13—H13B	109.1	C32—C37—H37A	109.5
N15—C14—C13	102.29 (19)	C32—C37—H37B	109.5
N15—C14—H14A	111.3	H37A—C37—H37B	109.5
C13—C14—H14A	111.3	C32—C37—H37C	109.5
N15—C14—H14B	111.3	H37A—C37—H37C	109.5
C13—C14—H14B	111.3	H37B—C37—H37C	109.5
H14A—C14—H14B	109.2	C34—C38—H38A	109.5
C11—N15—C31	123.7 (2)	C34—C38—H38B	109.5
C11—N15—C14	112.87 (19)	H38A—C38—H38B	109.5
C31—N15—C14	120.67 (18)	C34—C38—H38C	109.5
C22—C21—C26	122.7 (2)	H38A—C38—H38C	109.5
C22—C21—N12	118.5 (2)	H38B—C38—H38C	109.5
C26—C21—N12	118.8 (2)	C36—C39—H39A	109.5
C23—C22—C21	117.4 (2)	C36—C39—H39B	109.5
C23—C22—C27	120.8 (2)	H39A—C39—H39B	109.5
C21—C22—C27	121.8 (2)	C36—C39—H39C	109.5
C22—C23—C24	122.1 (2)	H39A—C39—H39C	109.5
C22—C23—H23	118.9	H39B—C39—H39C	109.5
C24—C23—H23	118.9	C42—C41—Ru	127.91 (17)
C25—C24—C23	118.6 (2)	C42—C41—H41	116.0
C25—C24—C28	120.9 (2)	Ru—C41—H41	116.0
C23—C24—C28	120.5 (2)	C43—C42—C47	117.5 (2)
C24—C25—C26	121.4 (2)	C43—C42—C41	118.7 (2)
C24—C25—H25	119.3	C47—C42—C41	123.7 (2)
C26—C25—H25	119.3	C44—C43—C42	121.0 (2)
C21—C26—C25	117.7 (2)	C44—C43—H43	119.5
C21—C26—C29	121.4 (2)	C42—C43—H43	119.5
C25—C26—C29	120.9 (2)	C45—C44—C43	121.5 (2)
C22—C27—H27A	109.5	C45—C44—H44	119.3
C22—C27—H27B	109.5	C43—C44—H44	119.3
H27A—C27—H27B	109.5	C44—C45—C46	119.0 (2)
C22—C27—H27C	109.5	C44—C45—H45	120.5
H27A—C27—H27C	109.5	C46—C45—H45	120.5
H27B—C27—H27C	109.5	C47—C46—C45	120.4 (2)
C24—C28—H28A	109.5	C47—C46—H46	119.8
C24—C28—H28B	109.5	C45—C46—H46	119.8
H28A—C28—H28B	109.5	C46—C47—C42	120.6 (2)
C24—C28—H28C	109.5	C46—C47—C48	117.4 (2)
H28A—C28—H28C	109.5	C42—C47—C48	122.0 (2)
H28B—C28—H28C	109.5	O49—C48—C47	125.4 (2)
C26—C29—H29A	109.5	O49—C48—H48	117.3
C26—C29—H29B	109.5	C47—C48—H48	117.3
H29A—C29—H29B	109.5	C48—O49—Ru	128.47 (15)
			
C41—Ru—C11—N12	−5.8 (3)	C14—N15—C31—C32	−81.5 (3)
O49—Ru—C11—N12	85.9 (2)	C11—N15—C31—C36	−106.7 (3)
Cl1—Ru—C11—N12	−95.3 (2)	C14—N15—C31—C36	93.5 (3)
Cl2—Ru—C11—N12	178.85 (15)	C36—C31—C32—C33	4.1 (4)
C41—Ru—C11—N15	170.79 (18)	N15—C31—C32—C33	179.0 (2)
O49—Ru—C11—N15	−97.46 (18)	C36—C31—C32—C37	−173.4 (2)
Cl1—Ru—C11—N15	81.27 (17)	N15—C31—C32—C37	1.4 (3)
Cl2—Ru—C11—N15	−4.6 (3)	C31—C32—C33—C34	−1.6 (4)
N15—C11—N12—C21	179.8 (2)	C37—C32—C33—C34	176.1 (2)
Ru—C11—N12—C21	−3.4 (4)	C32—C33—C34—C35	−1.2 (4)
N15—C11—N12—C13	0.3 (3)	C32—C33—C34—C38	179.8 (3)
Ru—C11—N12—C13	177.2 (2)	C33—C34—C35—C36	1.6 (4)
C11—N12—C13—C14	4.1 (3)	C38—C34—C35—C36	−179.5 (3)
C21—N12—C13—C14	−175.4 (2)	C34—C35—C36—C31	0.9 (4)
N12—C13—C14—N15	−6.4 (3)	C34—C35—C36—C39	−178.3 (2)
N12—C11—N15—C31	−166.3 (2)	C32—C31—C36—C35	−3.7 (4)
Ru—C11—N15—C31	16.3 (3)	N15—C31—C36—C35	−178.6 (2)
N12—C11—N15—C14	−5.1 (3)	C32—C31—C36—C39	175.4 (2)
Ru—C11—N15—C14	177.51 (17)	N15—C31—C36—C39	0.6 (4)
C13—C14—N15—C11	7.4 (3)	C11—Ru—C41—C42	106.4 (2)
C13—C14—N15—C31	169.2 (2)	O49—Ru—C41—C42	11.9 (2)
C11—N12—C21—C22	−91.5 (3)	Cl1—Ru—C41—C42	−165.7 (2)
C13—N12—C21—C22	87.9 (3)	Cl2—Ru—C41—C42	−76.1 (2)
C11—N12—C21—C26	91.2 (3)	Ru—C41—C42—C43	171.49 (18)
C13—N12—C21—C26	−89.4 (3)	Ru—C41—C42—C47	−8.6 (3)
C26—C21—C22—C23	−1.8 (3)	C47—C42—C43—C44	−0.2 (3)
N12—C21—C22—C23	−178.94 (19)	C41—C42—C43—C44	179.7 (2)
C26—C21—C22—C27	178.3 (2)	C42—C43—C44—C45	−0.5 (4)
N12—C21—C22—C27	1.1 (3)	C43—C44—C45—C46	0.7 (4)
C21—C22—C23—C24	0.9 (3)	C44—C45—C46—C47	−0.3 (4)
C27—C22—C23—C24	−179.2 (2)	C45—C46—C47—C42	−0.3 (4)
C22—C23—C24—C25	0.4 (4)	C45—C46—C47—C48	−179.0 (2)
C22—C23—C24—C28	−179.0 (2)	C43—C42—C47—C46	0.5 (3)
C23—C24—C25—C26	−0.9 (4)	C41—C42—C47—C46	−179.3 (2)
C28—C24—C25—C26	178.6 (2)	C43—C42—C47—C48	179.2 (2)
C22—C21—C26—C25	1.4 (3)	C41—C42—C47—C48	−0.7 (3)
N12—C21—C26—C25	178.5 (2)	C46—C47—C48—O49	179.8 (2)
C22—C21—C26—C29	−179.5 (2)	C42—C47—C48—O49	1.1 (4)
N12—C21—C26—C29	−2.4 (3)	C47—C48—O49—Ru	7.0 (3)
C24—C25—C26—C21	0.0 (3)	C41—Ru—O49—C48	−11.70 (19)
C24—C25—C26—C29	−179.1 (2)	C11—Ru—O49—C48	−109.79 (19)
C11—N15—C31—C32	78.3 (3)	Cl2—Ru—O49—C48	100.04 (18)

### Hydrogen-bond geometry (Å, °)

**Table:**

*D*—H···*A*	*D*—H	H···*A*	*D*···*A*	*D*—H···*A*
C29—H29C···Cl1	0.98	2.67	3.634 (3)	166
C39—H39C···Cl1	0.98	2.76	3.371 (3)	121
C37—H37A···O49	0.98	2.39	3.268 (3)	150
C13—H13B···Cl1^i^	0.99	2.94	3.395 (2)	109
C14—H14A···Cl1^i^	0.99	2.90	3.324 (3)	107
C27—H27A···Cl2^i^	0.98	2.85	3.724 (3)	149
C37—H37C···Cl2^i^	0.98	2.88	3.736 (3)	146
C25—H25···Cl1^ii^	0.95	2.98	3.831 (3)	150
C29—H29A···Cl1^ii^	0.98	2.71	3.673 (3)	169
C46—H46···Cl2^iii^	0.95	3.04	3.553 (2)	115
C48—H48···O49^iii^	0.95	2.50	3.014 (3)	114

Symmetry codes: (i) *x*, −*y*+1, *z*+1/2; (ii) −*y*+1, *x*, −*z*; (iii) −*x*, −*y*+1, *z*.
